# Toward precision psychiatry using HD-EEG and normative modeling

**DOI:** 10.1192/j.eurpsy.2025.288

**Published:** 2025-08-26

**Authors:** M. Hassan, A. Ebadi, A. Mheich, J. Tabbal, A. Kabbara, G. Robert, A. Lefebvre, A. Iftimovici, B. Rodríguez-Herreros, N. Chabane, S. Allouch

**Affiliations:** 1 MINDIG, Rennes, France; 2 School of Science and Engineering, Reykjavik University, Reykjavik, Iceland; 3 Service des Troubles du Spectre de l’Autisme et apparentés, Département de psychiatrie, Lausanne University Hospital (CHUV), Lausanne, Switzerland; 4 U1228 Empenn UMR 6074 IRISA, Rennes; 5 Paris Saclay University, Neurospin, CEA Saclay, Service Avis et Expertise TND, Fondation Vallée; 6 Université Paris Cité, Institute of Psychiatry and Neuroscience of Paris (IPNP), INSERM U1266, Team “Pathophysiology of psychiatric disorders”, GDR 3557-Institut de Psychiatrie; 7 GHU Paris Psychiatrie et Neurosciences, Pôle hospitalo-universitaire d’évaluation, prévention, et innovation thérapeutique (PEPIT), Paris, France; 8 Service des Troubles du Spectre de l’Autisme et apparentés, Département de psychiatrie, Lausanne University Hospital (CHUV), Lausanne, Switzerland

## Abstract

**Introduction:**

Electroencephalography (EEG) has been extensively studied for decades in psychiatric research. However, its integration into clinical practice as a diagnostic or prognostic tool remains unachieved. We hypothesize that a key reason for this is the underlying heterogeneity among patients, which is often overlooked in psychiatric EEG research that relies on a case-control approach.

**Objectives:**

The main objective of this study is to quantify the electrophysiological heterogeneity of psychiatric disorders.

**Methods:**

We combine HD-EEG with normative modeling to quantify this heterogeneity using two well-established and extensively investigated EEG characteristics—spectral power and functional connectivity—across a cohort of 1,674 patients with attention-deficit/hyperactivity disorder, autism spectrum disorder, learning disorder, or anxiety, and 560 matched controls, see figure 1.

**Results:**

Normative models revealed that deviations from population norms among patients were highly heterogeneous and frequency-dependent. The spatial overlap of deviations among patients did not exceed 40% for spectral power and 24% for connectivity. Taking individual deviations into account significantly enhanced comparative analysis and the identification of patient-specific markers, which showed a correlation with clinical assessments.

**Image 1:**

**Figure:**
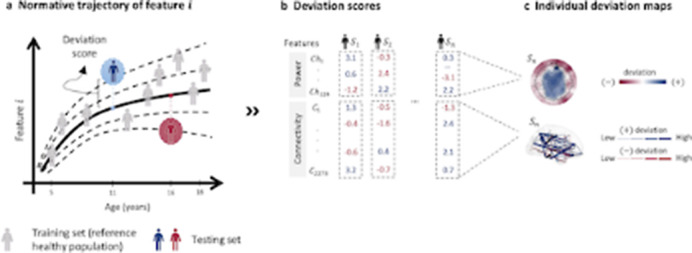

**Conclusions:**

Our study underscores the necessity of moving EEG research in psychiatry beyond the group-level approach to achieve precision psychiatry.

**Disclosure of Interest:**

None Declared

